# Supplementary material for: Phosphorylation-State Modulated Binding of HSP70: Structural Insights and Compensatory Protein Engineering
Source: bioRxiv. 2025 Feb 17:2025.02.17.637997. Preprint. [Version 1] doi: 10.1101/2025.02.17.637997 (PMC11870554; doi:10.1101/2025.02.17.637997)
Supplement: Supplement 1 [file media-1.pdf]

SUPPORTING INFORMATION

Supporting Figures

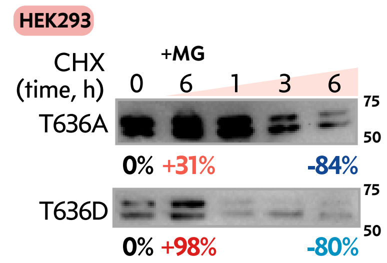

Figure S1. Steady-state levels of HSP70-T636A and HSP70-T636D in HEK-293 cells. Representative immunoblots of HSP70 steady-state levels with different amino acids at position 636 following the addition of cycloheximide (CHX) or MG132 with the percent change from time 0 at each 6 h time point. Cell extracts were separated via SDS-PAGE, and a Flag antibody detected the exogenous HSP70 levels.

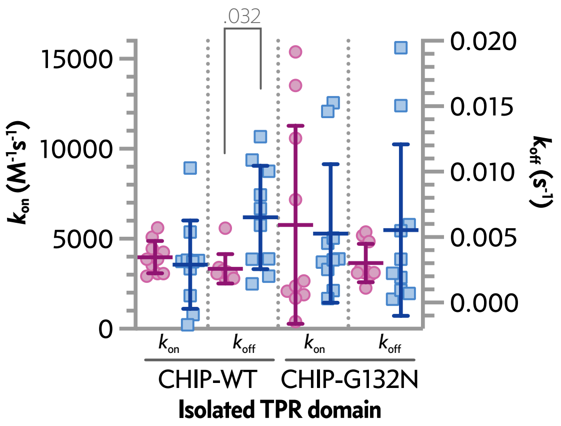

Figure S2. G132N recovers increased k_{off} between CHIP and pEEVD peptide. Analysis of G132N using biolayer interferometry for wild type (WT) and G132N-CHIP-TPR measured by k_{on} and k_{off}. Data represented by dot plot and summarized by the mean ± SD analyzed via two-way ANOVA: main effects of TRP construct, $F(1, 36) = 0.04545$, $P= 0.832$ and EEVD peptide, $F(1, 36) = 6.718$, $P = 0.014$, and an interaction effect between TPR construct and EEVD peptide, $F(1, 36) = 0.3208$, $P < 0.575$. TPR construct accounted for 0.11% of the total variation, while the EEVD peptide accounted for 16%. Post-test results of pairwise comparisons < 0.05 are included in the plot.

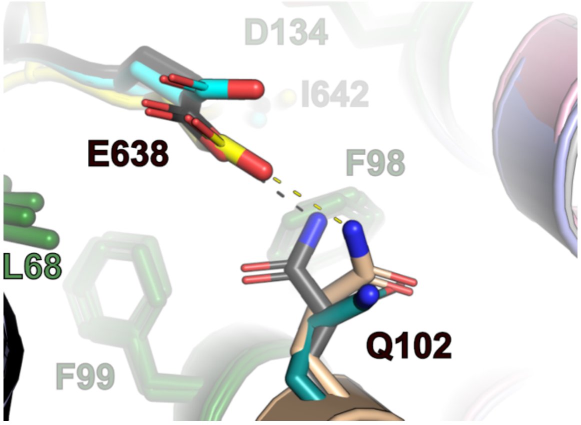

Figure S3. Enhanced detail of CHIP Q102 interaction with HSP70 E638. A close-up view of the differential interactions of HSP70 E638 and CHIP Q102. CHIP-TPR. Overlaid structures include CHIP-TPR in complex with the helical lid+tail domain of HSP70 (PDB ID 4KBQ: CHIP in light grey, HSP70 helical lid+tail domain in dark grey), in complex with EEVD (PDB ID 9DYA: CHIP in light blue, EEVD in yellow), and in complex with the pEEVD (9DYB: CHIP in light pink, pEEVD in cyan). Throughout, CHIP-TPR residues L68, F98, F99, and D134, which interact with EEVD, are colored forest green. The hydrogen bond O-N distance between the E638 side chain carboxylate and Q102 amide is 2.9 Å (black dashed line) for CHIP-TPR in complex with the helical lid+tail domain of HSP70 and 2.7 Å (yellow dashed line) for the CHIP-TPR in complex with EEVD. Movement of pEEVD and rotation of the CHIP-TPR Q102 side chain eliminates the hydrogen bond between the E638 side chain carboxylate and Q102 amide, with the O-N distance increasing to 5.8 Å.

Supporting Tables

Vector	Host	Experiment(s)	Tag	Key Mutation	Lab	AddgeneID
**CHIP-TPR**	Pro	Crystal Structures, Biolayer Interferometry	HIS	Wild-type	RCP	
**HSP70-lid/ tail**	Pro	Crystal Structures, Biolayer Interferometry	HIS	Wild-type	RCP	
**CHIP**	Pro	Fluorescent Polarization, In Vitro Ubiquitination	HIS	Wild-type	JCS	
**CHIP-K30A**	Pro	Fluorescent Polarization	HIS	K30A	JCS	
**CHIP- G132N**	Pro	Fluorescent Polarization, In Vitro Ubiquitination	HIS	G132N	JCS	
**CHIP TPR-CC**	Pro	Fluorescent Polarization, In Vitro Ubiquitination	HIS	UBOX deletion	JCS	
**CHIP TPR-UBOX**	Pro	Fluorescent Polarization, In Vitro Ubiquitination	HIS	CC deletion	JCS	
**CHIP CC-UBOX**	Pro	Fluorescent Polarization, In Vitro Ubiquitination	HIS	TPR deletion	JCS	
**CHIP TPR**	Pro	Fluorescent Polarization, In Vitro Ubiquitination	HIS	UBOX and CC deletion	JCS	
**WT HSP70**	Pro	In Vitro Ubiquitination	HIS	Wild-type	NGB	
**Empty Vector (pcDNA3)**	Euk	Co-Immunoprecipitation, Cell Proliferation	none	none	JCS	
**HSP70-WT**	Euk	Co-Immunoprecipitation, Cell Proliferation	FLAG	Wild-type	JCS	
**HSP70A**	Euk	Co-Immunoprecipitation, Cell Proliferation	FLAG	T636A	JCS	
**HSP70D**	Euk	Co-Immunoprecipitation, Cell Proliferation	FLAG	T636D	JCS	
**WT CHIP**	Euk	Co-Immunoprecipitation, Cell Proliferation	MYC	Wild-type	JCS	
**K30A CHIP**	Euk	Co-Immunoprecipitation, Cell Proliferation	MYC	K30A	JCS	
**G132N CHIP**	Euk	Co-Immunoprecipitation, Cell Proliferation	MYC	G132N	JCS	
**LgBiT-HSP70-WT**	Euk	NanoBiT	LgBiT Nanoluc	Wild-type	KMS	
**LgBiT-HSP70-A**	Euk	NanoBiT	LgBiT Nanoluc	T636A	JCS	
**LgBiT-WT HSP70-D**	Euk	NanoBiT	LgBiT Nanoluc	T636D	JCS	
**SmBiT-CHIP-WT**	Euk	NanoBiT	SmBiT Nanoluc	Wild-type	KMS	
**SmBiT-CHIP-K30A**	Euk	NanoBiT	SmBiT Nanoluc	K30A	JCS	
**SmBiT-CHIP-G132N**	Euk	NanoBiT	SmBiT Nanoluc	G132N	JCS	

Table S1: Listed are the vectors used in the study. The experimental use, length, tag, mutation, and where the construct was acquired from are included for each construct. Vector sequences are available via Addgene. KMS = Kenneth Matt Scaglione, Duke University (matt.scaglione@duke.edu).

	HsCHIP$_{21\text{-}154}$ + HsHsp70$_{634\text{-}641}$	HsCHIP$_{21\text{-}154}$ + pT$_{636}$–HsHsp70$_{634\text{-}641}$
**Data Collection**		
Beam Line	ALS 4.2.2	ALS 4.2.2
Wavelength (Å)	1.0000	1.0000
Space group	$C222_1$	$C222_1$
**Cell dimensions**		
a, *b*, *c* (Å)	46.89, 74.36, 77.63	37.58, 45.93, 78.15
a, *b*, *g* (°)	90, 90, 90	90, 90, 90
Resolution (Å)[a]	39.67-1.89 (1.98-1.89)	39.60-1.59 (1.64-1.59)
R_{merge}[b]	0.170 (1.014)	0.092 (0.859)
R_{meas}[c]	0.198 (1.21)	0.107 (1.013)
$CC_{1/2}$	0.988 (0.442)	0.997 (0.612)
$<I/sI>$	7.05 (0.99)	10.32 (1.25)
Wilson *B* factor (Å^2)	14.66	13.58
Completeness (%)	98.93 (91.46)	99.70 (97.38)
Redundancy	3.8 (3.2)	3.7 (3.5)
No. of reflections	77,973 (7,819)	127,365 (8,914)
No. of unique reflections	20,723 (2,406)	34,506 (2,570)
**Refinement**		
No. reflections for refinement	11,040 (1,242)	18,531 (1,855)
R_{work}/R_{free}	0.189 / 0.234	0.173 / 0.213
**Average *B* factors**		
Protein	18.18	16.53
Water	24.47	24.95
Ions	21.11	20.47
**R.m.s deviations**		
Bond lengths (Å)	0.010	0.006
Bond angles (°)	1.160	0.780
**Ramachandran plot statistics**		
Favored regions % (#)	99.2 (131/132)	97.9 (137/140)
Allowed regions % (#)	100.0 (132/132)	100.0 (140/140)
Disallowed regions	0.0	0.0
**MolProbity validation statistics**		
Cb deviations >0.25Å	0	0
MolProbity clash score	1.85	1.80
MolProbity clash percentile	99th percentile (N=1,784, all resolutions)	99th percentile (N=1,784, all resolutions)
MolProbity score	0.95	1.00
MolProbity score percentile	100th percentile (N=27,675, all resolutions)	100th percentile (N=27,675, all resolutions)
PDB ID	9DYA	9DYB

Table S2: X-ray data collection and structure refinement used to generate Figure 3. [a] Values in parentheses are for the highest resolution shell. [b] The merging *R* factor measures the consistency between multiple measurements of the same reflection. [c] The corrected *R* factor was the final adjustment used for known systematic errors in the data.

Name	Target	Type	Company	Reference Number
DYKDDDDK Tag (D6W5B)	FLAG-Tagged HSP70	Rabbit mAb (HRP conjugate)	Cell Signaling	86861S
Myc-Tag (9B11)	MYC-Tagged CHIP	Mouse mAb (HRP conjugate)	Cell Signaling	4040S
CHIP (C3B8)	CHIP	Rabbit mAb	Cell Signaling	2080S
Hsp70/Hsp72 (C92F3A-5)	HSP70	mAb	Enzo	ADI-SPA-810

Table S3: Antibodies used in the study.

Figure	Type/Antibody	Image
1I	IP: Stain-free	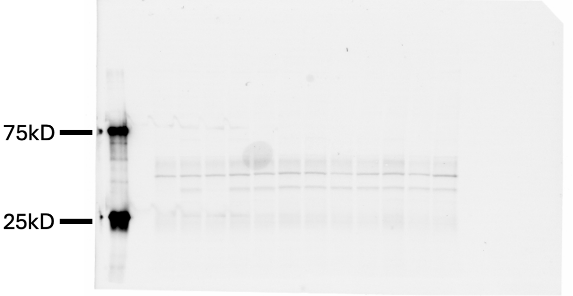
	IP:Flag	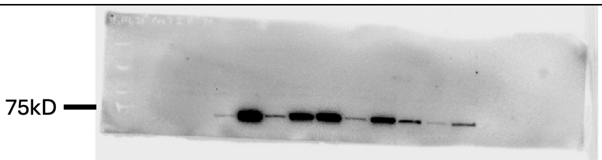
	IP:MYC	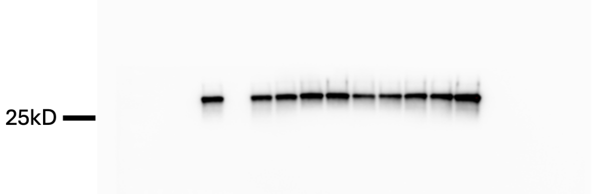
	Input: Stain-free	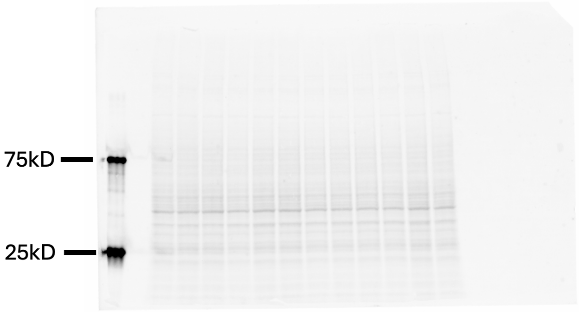
	Input: Flag	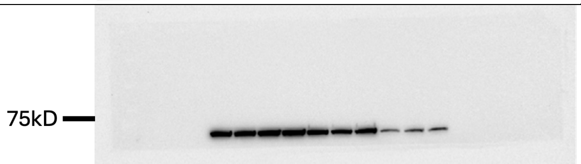
	Input: MYC	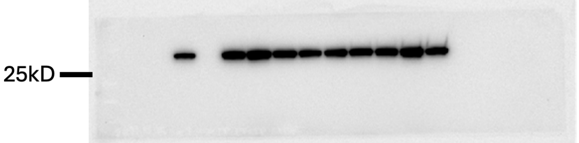

**2J**	Stain-free	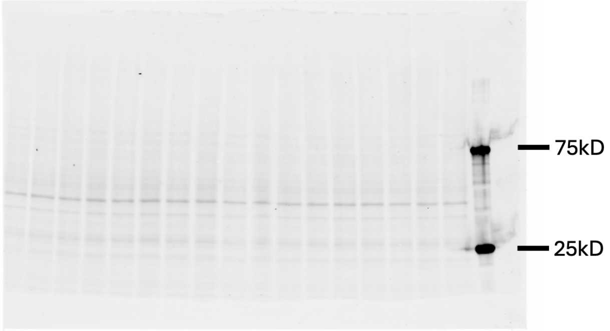
	FLAG	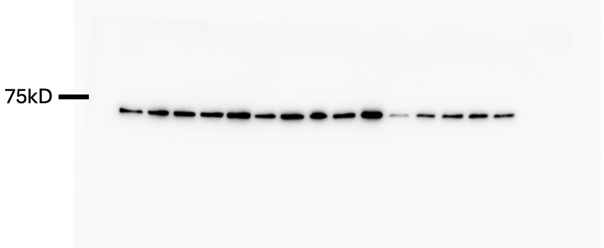
**2K**	Stain-free	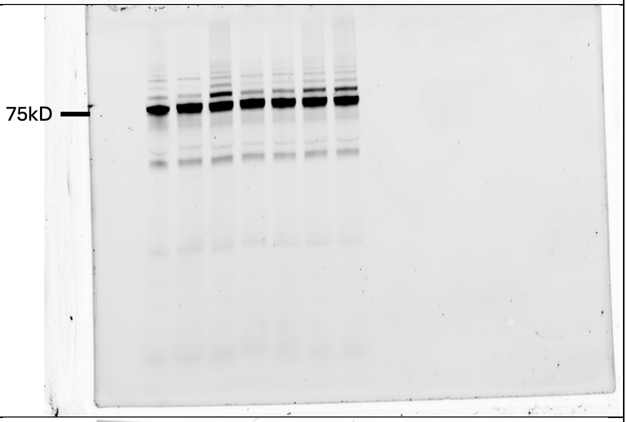
**S1**	Stain-free	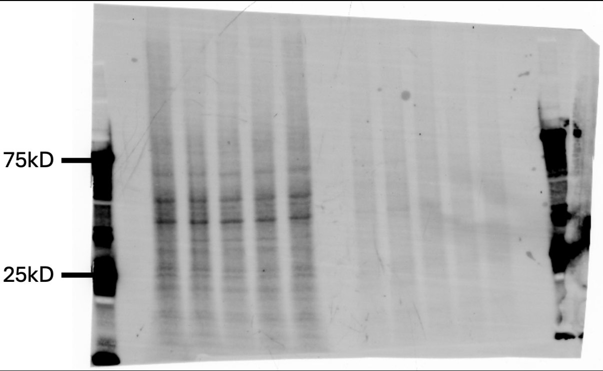

**S1A** (cont.)	Flag	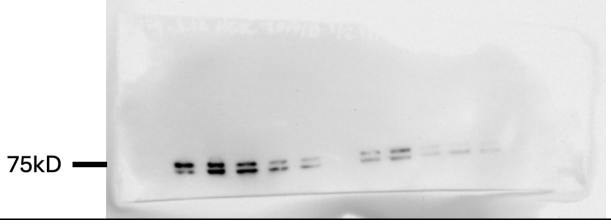

Table S4. Stain-free and unmodified immunoblots. The stain-free membrane before blocking and primary antibody and unmodified representative immunoblots are included for each figure that utilizes an immunoblot. The antibody used is included in the second column.
