# Supplementary material for: Phosphorylation-State Modulated Binding of HSP70: Structural Insights and Compensatory Protein Engineering
Source: bioRxiv. 2025 Feb 17:2025.02.17.637997. Preprint. [Version 1] doi: 10.1101/2025.02.17.637997 (PMC11870554; doi:10.1101/2025.02.17.637997)
Supplement: Supplement 2 [file media-2.pdf]

TABLES

	HEK-293		COS-7	
**Source of Variation**	**% of total variation**	**P value**	**% of total variation**	**P value**
CHIP	0.07	0.997	0.12	0.993
Time	10	8.57E-03	31	2.27E-05
HSP70	0.04	0.873	0.04	0.869
CHIP x Time	0.10	0.995	0.01	1.000
CHIP x HSP70	0.02	1.000	0.09	0.996
Time x HSP70	0.01	0.920	0.23	0.688
CHIP x Time x HSP70	0.18	0.988	0.07	0.997

Table 1. Three-way ANOVA on CHIP and HSP70 modifications and time with cell counts. The table shows the percentage of total variation and P values for each source of variation, including single terms and interaction terms (denoted by 'x'), for both HEK-293 and COS-7 cell lines.
